# *In vitro* models to study natural killer cell dynamics in the tumor microenvironment

**DOI:** 10.3389/fimmu.2023.1135148

**Published:** 2023-06-28

**Authors:** Valentina Carannante, Martin Wiklund, Björn Önfelt

**Affiliations:** ^1^ Department of Applied Physics, Science for Life Laboratory, KTH Royal Institute of Technology, Stockholm, Sweden; ^2^ Center for Infectious Medicine, Department of Medicine Huddinge, Science for Life Laboratory, Karolinska Institutet, Stockholm, Sweden

**Keywords:** NK cells, tumor microenvironment, tumor spheroids, tumor organoids, microscopy, flow cytometry, tissue sectioning, live cell imaging

## Abstract

Immunotherapy is revolutionizing cancer therapy. The rapid development of new immunotherapeutic strategies to treat solid tumors is posing new challenges for preclinical research, demanding novel *in vitro* methods to test treatments. Such methods should meet specific requirements, such as enabling the evaluation of immune cell responses like cytotoxicity or cytokine release, and infiltration into the tumor microenvironment using cancer models representative of the original disease. They should allow high-throughput and high-content analysis, to evaluate the efficacy of treatments and understand immune-evasion processes to facilitate development of new therapeutic targets. Ideally, they should be suitable for personalized immunotherapy testing, providing information for patient stratification. Consequently, the application of *in vitro* 3-dimensional (3D) cell culture models, such as tumor spheroids and organoids, is rapidly expanding in the immunotherapeutic field, coupled with the development of novel imaging-based techniques and -omic analysis. In this paper, we review the recent advances in the development of *in vitro* 3D platforms applied to natural killer (NK) cell-based cancer immunotherapy studies, highlighting the benefits and limitations of the current methods, and discuss new concepts and future directions of the field.

## 1 Introduction

Immunotherapy comprises a large set of therapeutical strategies aimed at using or improving immune cell activity against tumors. Natural killer (NK) cells are NKp46^+^ innate lymphocytes that participate in cancer immune surveillance, by eliminating tumor cells by cell-mediated cytotoxicity and pro-inflammatory cytokine release (1). Our ability to design efficient NK-based therapies requires broad knowledge of NK cell behavior in the tumor environment (TME) and screening platforms that reproduce such environments *in vitro*. NK cell activity has mostly been studied using two-dimensional (2D) cultures *in vitro*, and mouse models *in vivo*. These models have provided invaluable information in terms of phenotypic and functional characterization of NK cells. However, they present multiple limitations in terms of translational potential.

2D cell cultures are frequently used in research since they are easy to handle and well compatible with wide range of assays, especially if they involve cells growing in suspension, such as lymphocytes. However, a vast proportion of cells constituting human tissue are adherent. Generally, oxygen plasma-treated polystyrene surfaces in combination with serum-supplemented cell culture media are used to support cell adhesion and maintenance. Other materials that have been used in the microfluidic field and also demonstrated to be suitable for adherent cell growth include polydimethylsiloxane (PDMS) (2), cyclo-olefin polymer (3), polymethyl-methacrylate (4) and polycarbonate (5). Independently of the material used, adherent cells that interact with flat surfaces tend to distribute as monolayers. But cell monolayers are far from being representative of the three-dimensional (3D) architecture of the original tissue. The third dimension matters as the function of a cell in a tissue is dependent on its position in relation to the extracellular matrix (ECM) and the surrounding cells (6, 7). The composition of the ECM, cell-to-cell adhesion and mechanical stress contribute to cell proliferation, differentiation, and migration (8–11). The gradients of gas and nutrients in a tissue determine cell fate, shaping their metabolic and apoptotic programs (12–14). Chemotactic gradients modulate the direction and dynamics of cell migration (15). This combination of biomechanical and biochemical cues present in the tissues might have significant implications for NK cell-mediated tumor surveillance and response to treatment (16, 17). Since 2D cell cultures show multiple limitations in reproducing the original features of human tissues (18), their use for research is usually complemented with mouse models, such as genetically engineered mouse models, patient-derived tumor xenografts and humanized mice. Mouse models can provide valuable information on the aetiology and the progression of diseases, as well as safety of therapies, but they are not optimal models for immunotherapy screening and precision medicine. Mouse models are also very expensive, not suitable for high-throughput testing, and they do not fully recapitulate the stromal composition of human tissue.

**Figure 1:**
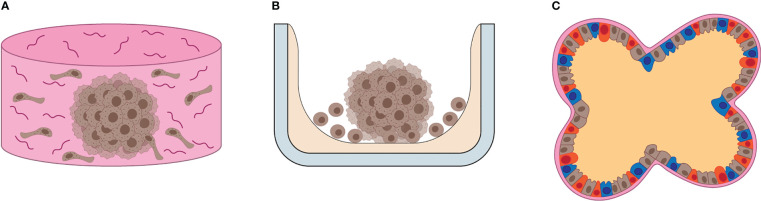
Overview of 3D cultures. **(A)** Illustration of scaffold-based 3D cell culture. Scaffolds resemble the ECM composition and 3D architecture of human tissues, providing support for cells to grow and differentiate *in vitro*. **(B)** Illustration of scaffold-free 3D cell culture. Often the substrate (gray) is coated by a non-adhesive, inert chemical (beige), which promotes cell-to-cell interaction, ECM and growth factor release, leading to the formation of self-sustained 3D cell cultures *in vitro*. **(C)** The illustration depicts the general features of organoids, such as multicellular composition, defined cellular distribution, cell differentiation and basolateral specialization of functions, such as EMC production in the basal area and fluid release in the apical area of cells.

To overcome these limitations, a wide variety of techniques have been developed and optimized for routine use of 3D cultures in biological research (18–21). 3D cultures are commonly categorized into scaffold-based and scaffold-free cultures. In scaffold-based 3D cultures, a substrate is provided to simulate the properties of ECM and promote cell adhesion (**Figure 1A**) (22–28), and they are commonly applied in bone and myocardial tissue regeneration (29). Scaffold-free 3D cultures rely on the formation of multicellular aggregates by promoting the adhesion of cells to each other rather than to a substrate (**Figure 1B**). Scaffold-free 3D cultures are low-cost and they guarantee high levels of reproducibility (18). Their main applications include studies of solid tumor models, immune cell-solid tumor interaction, drug screening and formation of organotypic models. The term “spheroids” generically refers to tight cellular aggregates with spherical shape (**Figure 1B**). They can be composed of multiple cell types (heterotypic spheroids) or a single cell type (homotypic spheroids). The term “organoids” usually refers to 3D cultures composed of multiple cell types with specific localization and roles within the aggregates, resembling the composition, organization and function of the original tissue (30). Such specialization within the organoids is usually obtained driving the differentiation of stem cells (induced-pluripotent stem cells (iPS), primary stem cells, adult stem cells) *in vitro*. In this regard, organoids are effectively small reproductions of organs *in vitro* (**Figure 1C**).

The boundaries between “spheroids” and “organoids” become less clear in the context of tumors, i.e. tissue that by definition lose structural and functional organization (31). For simplicity, we will collectively refer to 3D models used to recapitulate tumor tissue as “tumor spheroids”. In the following sections, we describe the currently available methods to characterize NK cell phenotype, cytotoxicity and infiltration in tumor spheroids, as well as their application in immunotherapy testing. The application of tumor spheroids in the NK cell field goes back to the late 70´, their use remained sporadic until very recently. Nowadays, the development of new tools for culture and data analysis is boosting the application of tumor spheroids in NK cell research.

## 2 Natural killer cells

NK cells are innate lymphocytes that promote immune surveillance and tissue homeostasis (32). Fast activation is one of the key features of NK cells, which contributed to their discovery: in 1975, R. Kiessling, E. Klein and H. Wigzell reported their identification of “naturally occurring lymphocytes” able to “spontaneously kill” mouse Moloney leukemia cells (33, 34). The term “spontaneously” referred to their ability to be cytotoxic *in vitro* within an hour, without requiring prior exposure to the same tumor cells (33, 34). Rapid effector functions and lack of prior sensitization markedly distinguishes NK cells from T cells, that require 6 hours (with co-stimulation) up to 30 hours (in the absence of co-stimulation) to be fully activated (35). Cell-mediated cytotoxicity and pro-inflammatory cytokine release are the most prominent NK cell effector functions (36). However, multiple NK cell subsets with varying functions have been reported since their discovery.

NK cells express the cell surface marker NKp46, and they can be found in the body as circulating or tissue-resident cells (37). Circulating NK cells, also called “conventional” NK cells, represent 5-15% of peripheral blood mononuclear cells (PBMCs). They constantly travel between spleen, lymph nodes and inflamed tissues using blood and lymphatic vessels (38). Tissue-resident NK cells can be found in the liver, lung, adipose tissue, and uterus during pregnancy (39).

Two main NK cell subsets have been characterized in humans: CD56^Bright^ CD16^-^ and CD56^Dim^ CD16^+^ (40). CD56^Bright^ CD16^-^ NK cells are preferentially distributed in lymph nodes, tonsils, and uterus, and they show strong cytokine production (1, 41). CD56^Dim^ CD16^+^ cells represent approximately 90% of circulating NK cells in blood and spleen, and their main features are cytotoxic activity and cytokine production (42). CD56^Bright^ CD16^-^ NK cells express high levels of CCR7 and L-selectin, while both receptors are absent on CD56^Dim^ NK cells (43, 44). On the other hand, CD56^Dim^ CD16^+^ NK cells present high levels of CXCR1, CX3CR1 and Chem23, chemokine receptors that drive their recruitment into inflamed tissues (45–47). Migration into peripheral tissues is assisted by additional adhesion proteins, such as low-affinity ligands for E-selectin and P-selectin, that promote leukocyte rolling on the vascular bed, and high-affinity integrins that mediate firm adhesion to endothelial cells and subsequent trans-endothelial migration (1).

**Figure 2:**
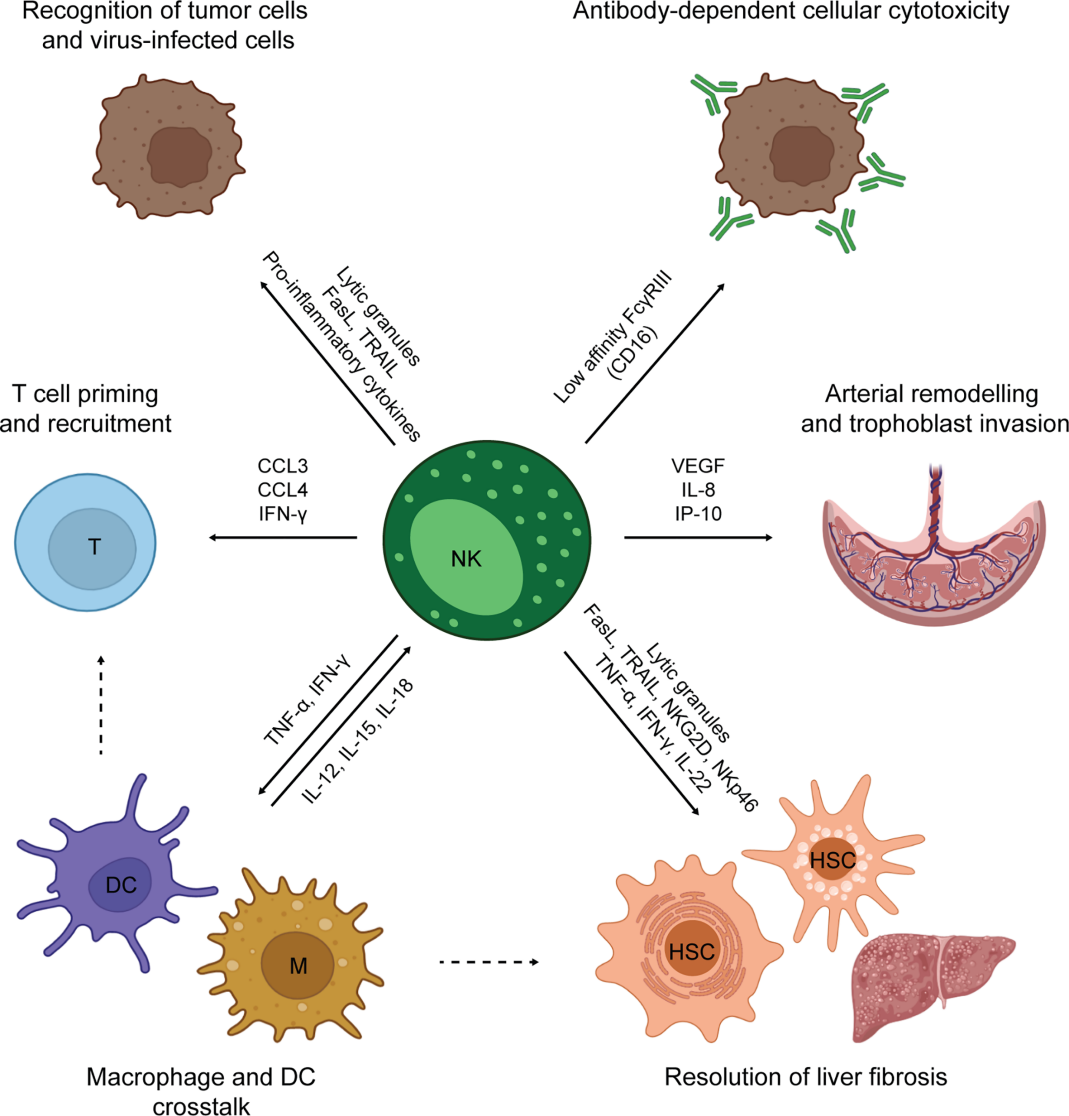
Overview of NK cell functions.

NK cytolytic activity is mediated by both release of perforin/granzyme granules and death receptor activation (**Figure 2**) (48). Cytokines produced by NK cells comprise pro-inflammatory mediators, such as interferon γ (IFN-γ) and tumor necrosis factor-α (TNF-α), and immunosuppressive mediators, such as interleukin (IL)-10. NK cells also secrete growth factors, such as granulocyte macrophage colony-stimulating factor and granulocyte colony-stimulating factor (45), and several chemokines, including CCL2, CCL3, CCL4 and CCL5 (49). In addition, NK cells can perform antibody-depended cellular cytotoxicity (ADCC). ADCC is a potent type of cell-mediated cytotoxicity that relies on antibody crosslinking on the target cell (1). At early phases of immune responses, NK cells produce IFN-γ, promoting the immunoglobulin isotype switch towards IgG in B cells. At late phases of the immune responses and during antigen re-challenge, NK cells use the CD16a receptor, also called FcγRIIIa, to recognize target cells covered with IgG immunoglobulins. Such recognition induces a strong release of granzyme and perforin, that ultimately kills the target cell. ADCC also represents an example of cross-talk between NK cells and adaptive immunity.

NK cell activation is regulated by a dynamic system of inhibitory and activating signals. This system mainly relies on cell surface receptors that discriminate between healthy and unhealthy cells (36). Thus, tumor cells become susceptible to NK cells due to increased expression of activating ligands and/or decreased expression of inhibitory ligands (50). The main NK cell activating receptors are CD16, NKG2D, DNAM-1 and the natural cytotoxicity receptors (NCRs) NKp30, NKp44 and NKp46 (51), while CD94/NKG2A (C-type Lectin superfamily glycoproteins) (51), TIGIT (52, 53), LAG-3 and TIM-3 are inhibitory receptors. In addition, NK cells express KIR receptors, which are encoded by the KIR polygenic and polymorphic locus that includes both activating and inhibitory variants. Two KIRs haplotypes have been characterized in humans, A and B, differing in the amount of inhibitory and activating KIRs, respectively (54). KIR receptors recognize the ubiquitously expressed HLA class I molecules (HLA-A, HLA-B, HLA-C). NK cells constantly undergo a process of education that finely tunes their responsiveness, optimizing their ability to mount an immune response against cells with reduced HLA class I expression (typically virus-infected and tumor cells), while maintaining tolerance to self (healthy tissues expressing HLA class I) (55–57). NK response is potentiated by co-stimulatory receptors, such as CD2 and 2B4, and cytokines, such as IL-2, IL-15, IL-12, IL-18 and IL-21 (58). Cytokine exposure, as well as receptor activation, can trigger the differentiation of memory NK subsets, defined as long-lived, self-renewing NK cells showing enhanced effector function and proliferative capacity during secondary exposure to pathogens (59).

The NK cell activities described so far are mainly pro-inflammatory. However, NK cells also play a major role in tissue homeostasis (**Figure 2**) (39). Such a role is well-exemplified by decidual NK cells (dNK). dNK cells are poorly cytotoxic tissue-resident NK cells found in pregnant endometrial tissue to orchestrate placenta development. First, dNK cells produce angiogenic factors to re-model the uterine arterial system (60, 61). Secondly, they control extra-villous trophoblast cell invasion of spiral arteries, releasing IL-8 and interferon-inducible protein-10 (IP-10) (62). Both steps are crucial for ensuring a positive outcome of pregnancy (63). NK cells exist also in the adult liver and adipose tissues. After liver damage, NK cells collaborate with macrophages and hepatic stellate cells (HSC) in the resolution of fibrosis. In addition, cytotoxic NK cells kill hepatic stellate cells, specifically discerning between quiescent and activated cells (64). In adipose tissue, NK can sustain a local Th1 response and contribute to obesity-associated metabolic disease (65). NK cells are also involved in the prevention of autoimmunity, being able to kill immature dendritic cells (DCs) (66) and activated T cells (67, 68). In this review, we have primarily focused on the use of spheroids cultures to study the role of NK cells in cancer immune surveillance. However, multiple 3D models have been developed to study dNK cells and their role in pregnancy (69–74).

NK cells are key players in cancer immune surveillance and a variety of NK cell-based therapies are currently being developed and tested in Phase I/II clinical trials. Their ability to perform ADCC is taken into consideration while designing antibody-based immunotherapeutic strategies, and a plethora of activating receptors and co-receptors are used to produce new chimeric antigen receptor (CAR)-NK products for cellular immunotherapy. Compared to T cells, NK cell-based therapies show a better safety profile, rarely inducing severe adverse effects such as cytokine release syndrome and neurotoxicity. In addition, NK cells are not involved in graft-versus-host disease reactions, allowing the development of cell products from multiple sources, such as allogenic donors, cell lines and iPS cells, without safety concerns. Despite being effective in the treatment of hematopoietic cancers, the efficacy of NK cells in solid tumors is dramatically suppressed by the TME. For instance, presence of hypoxia (75) and acidic pH (76) affect NK cell survival and activity in the solid tumors (76–81). Tumor cells and fibroblasts cooperate in producing soluble factors that directly suppress NK cells, such as TGF-β (82), indoleamine 2,3-dioxygenase (83), adenosine and prostaglandin E2 (84). Expression of NK receptors and/or their corresponding ligands is often affected by the tumor microenvironment leading to decreased tumor recognition by NK cells (85–87).

To better understand, predict and possibly target NK cell inhibition in the tumor microenvironment, tumor spheroid cultures that mimic solid cancers have been developed and adopted to NK cell research. In the next paragraphs, we will describe the currently available methods for spheroid formation applied to NK cell studies.

## 3 Methods of spheroid formation applied to NK cell studies

### 3.1 Spinner cultures

**Figure 3:**
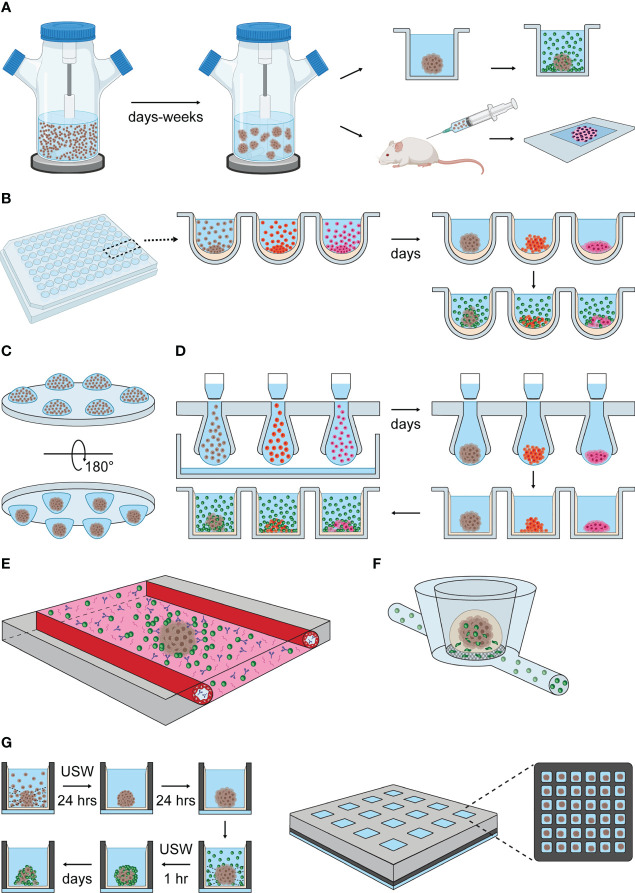
Methods of spheroid formation applied to NK cell research. **(A)** Spinner cultures. Tumor cell suspensions are transferred into spinner flasks that provide constant agitation and promote cell aggregation. Spheroid formation is reached within days or a few weeks, depending on the adhesive properties of each cell type. Spheroids generated by spinner cultures have been exposed to allogenic lymphocyte cultures (upper panel to the right) and implanted in allogenic mice (lower panel to the right) to study NK cell infiltration and clearance of tumors (88–91). **(B)** Liquid-overlay method. Cell suspensions are seeded into standard culture plates pre-treated with non-adhesive coatings, such as agarose or poly-HEMA, that indirectly promote spheroid formation by preventing cell adhesion to the plate. Gravity and cell confinement in wells facilitate cell aggregation (spheroids are obtained within a few days). NK cells can be co-cultured with tumor spheroids to study their infiltration and cytotoxic capacity *in vitro*. **(C)** Hanging-drop cultures. Drops of cell suspension is dispensed onto a standard culture plate, that is turned upside-down. Surface tension and gravity enforce the formation of a single spheroid per drop within a few days. **(D)** Illustrations of hanging-drop platforms compatible with long-term and high-throughput spheroid cultures, based on the general features of 3D Biomatrix and InSphero plates. Multiple drops are formed by transferring cell suspension into the inlets of an array plate. Liquid reservoirs prevent drop evaporation. After spheroid formation, each drop is displaced into a single well of an ULA plate by pipetting additional medium on top of the inlets or by centrifugation. From this point, the hanging drop-derived spheroids can be used as described in the liquid-overlay section. **(E)** Schematic representation of the microfluidic platform developed by Ayuso et al. (92). The microfluidic device consists of a central chamber filled with collagen type I hydrogel, hanging drop-derived spheroids and NK cells. Antibody solutions are perfused through two lateral channels covered with endothelial cells. **(F)** Schematic representation of the MIVO device developed by Marrella et al. (93, 94). Alginate-derived spheroids are transferred into the top chamber of the MIVO device, which resembles a trans-well insert. NK cell suspensions are perfused through capillaries running under the chambers containing spheroids, allowing NK cells to migrate through the trans-well membrane and infiltrate the tumor spheroid (95). **(G)** USW-induced spheroid formation in microwell chip. Left panel: cells are seeded into a glass-bottom microwell array chip coated with non-adhesive coating. USW exposure induce the formation of single spheroids in the center of each well. This step is followed by a period of spheroid stabilization in the absence of USW. Once spheroids are formed, NK cells are seeded into the wells under USW exposure to promote NK cell-spheroid interaction. The characterization of NK cell infiltration and killing in tumor spheroids can be performed directly in the chip by imaging, or off-chip by retrieving the samples from the chambers. Right panel: illustration of the multichambered microwell chip with 16 chambers, each containing 36 microwells, giving a total of 576 microwells per chip.

The application of spheroid cultures in the immunology field began in the late 70´ as models to study tumor allografts. Few methods of spheroid formation were available at that time, and the “spinner cultures” were among those. The method is based on culturing tumor cells in spinner flasks at constant stirring rates, promoting cell-to-cell interaction while preventing cell adhesion to the bottom of the flask (**Figure 3A**) (96). Sutherland et al. established mouse mammary sarcoma spheroids incubating cells in spinner flasks for 3-4 weeks (88). The spheroids were then exposed to allogenic mixed lymphocyte cultures (88) or implanted in the peritoneal cavity of allogenic mice (89–91) to study the effect of alloreactivity and immunization in solid tumors (88, 90). The spheroid implants could then be recovered to characterize the composition and the rate of immune cell infiltration (89, 91, 97). Similarly, Iwasaki et al. used the method to study the infiltration and cytolytic capacity of lymphokine activated killer (LAK) cells in human malignant glioma spheroids (98).

The spinner culture method allows great flexibility in terms of number of spheroids obtained, since different quantities can be produced simply scaling up or down the number of cells seeded and the volume of culture medium. The method is also easy-to-use and cost-effective. Areas of intense cell proliferation and necrosis, as well as cell migration and cell-to-cell interaction were detected within the spinner culture spheroids by image analysis (96, 98–100), making the method suitable to study tumor progression *in vitro*. In addition, proteomic profiling of glioma spinner cultures revealed substantial differences compared to monolayer cultures in terms of metabolism, antigen presentation and HLA-E expression, allowing He et al. to study the role of NKG2A in tumor resistance to NK cell therapy (101).

Nowadays, the application of spinner cultures to NK cell studies is quite rare, due to the limitations of the method. Cell aggregation is simply driven by cell-to-cell collision, limiting the use of the spinner cultures to cell types that adhere to each other without additional support. The incubation time is cell type-dependent, and a large variation of spheroid size can be observed within the same experiment, while many applications require homogeneously shaped and sized spheroid. In addition, the optimal stirring rate to prevent cell adhesion to the flask bottom might differ across cell types, requiring optimization steps for each experimental setup. Lastly, the process of spheroid formation cannot be monitored directly due to the constant agitation, and the spheroids need to be transferred to a different plate or substrate to be visualized by microscopy.

### 3.2 Liquid-overlay method

A spheroid-formation method that addresses some of the limitations of spinner cultures is the liquid-overlay technique (102–104). This technique involves covering cell culture surfaces with a thin layer of non-adhesive coating, preventing cell attachment to the substrate, and therefore favoring cell-to-cell interaction (**Figure 3B**) (103). Cell suspensions are simply transferred on top of the coated plates, and stable spheroid formation is obtained in a few days (102, 104). The thin and the transparent coating allows direct assessment of spheroid formation with standard transmission and fluorescence microscopes, reducing the sample handling steps. The use of the coating itself reduces the optimization steps required in the spinner cultures to set the proper spinning rate for each cell type.

The mostly used non-adhesive coating is the agarose gel, obtained by dissolving agarose powder in distilled water or buffers (phosphate buffer solution, cell culture medium) at concentrations usually spanning from 0.5% to 1.5% w/v (weight/volume) (104–106). Another commonly used non-adhesive coating is poly-2-hydroxyethyl methylacrylate (poly-HEMA) dissolved in ethanol (107). Apart from the chemical composition, agarose and poly-HEMA gels also differ in preparation time and long-term stability. The preparation of poly-HEMA plates requires three days (107), while agarose coating can be performed in few hours (104). However, poly-HEMA coated plates can be stored long-term at room temperature, while agarose gels are less stable, and degradation might occur over long-term cell cultures (108). In the last decades, multiple coatings have been developed that are commercially available as liquid solutions or through buying pre-coated culture plates. An example is represented by the ultra-low attachment (ULA) plates. ULA-plates are cell culture plates pre-coated with hydrophilic and neutrally charged hydrogel that prevents cell adhesion to the plastic. They are single-use and available in multiple formats.

Similar to spinner cultures, spheroids generated with the liquid-overlay techniques show necrotic (104–106, 109) and hypoxic (110) cores, deposition of ECM rich in collagen (I, III, IV, V) (111), fibronectin and laminin (112–114), and cell-to-cell and cell-to-matrix communication (105). These features make the liquid overlay-derived spheroids biologically relevant *in vitro* models. The method has been used to characterize the cytolytic activity and infiltration of NK cells in human glioma spheroids (115, 116), correlating NK activity with morphological and physical changes of the spheroids, such as loss of surface coherence (115), formation of cytoplasmic blebs (111), chromatin condensation (111, 116) and weight variation (117). It has also been used to identify adhesion molecules (112), receptors (106, 112, 114, 118–120) and metabolic pathways (121) involved in NK cell infiltration and cytotoxicity, as well as to test therapeutic strategies that enhance NK cell activity (105, 110, 120, 122–128). Importantly, the liquid-overlay technique is compatible with the use of primary material (124, 129–131), allowing the maintenance of tumor tissue explants up to a few weeks *in vitro*, retaining connective tissue components, endothelial cells, macrophage-like cells and blood capillaries (116).

The simplicity and scalability of the liquid-overlay technique contributed to the vast application of this method to multiple areas of biology research, especially immunology. However, the method still presents some flaws. Compared to spinner flasks, tumor cells are subjected to even less forces inducing cell-to-cell interaction, as the method solely relies on cell self-aggregation in the absence of substrate anchorage. Accordingly, a substantial number of solid tumor-derived cell lines fail to form anything but loose cell aggregates rather than compact spheroids in the liquid-overlay platform (102, 104, 107). Interestingly, very low success rate of spheroid formation is observed using healthy, non-cancerogenic cells (102, 104).

The ECM is essential player in cell-to-cell adhesion (132), and the quantity and the quality of ECM production by various cell types might influence their ability to form spheroids. Accordingly, the addition of ECM components to the liquid-overlay cultures has been shown to increase the spheroid tightness in some tumor models (107). Another strategy to improve the success rate of spheroid formation is to use U-bottom multi-well plates: gravity forces combined with the U-shaped well geometry promote cell sedimentation in the middle of the well, increasing the probability of cell-to-cell interactions. This strategy addresses the issue of multiple and heterogeneous spheroid formation, as single and homogeneous spheroids per well are more frequently obtained compared to other plate formats. In addition, spheroid confinement in a single well facilitates the detection of qualitative and quantitative spheroid changes over time by live-imaging. However, the U-shape and the optical properties of the commonly used plastic surfaces are not compatible with high-quality and high-resolution imaging, and sample transferring is still required for detailed analysis of biological processes, possibly affecting the integrity of the sample.

### 3.3 Hanging-drop method

The hanging-drop method is another popular technique for spheroid formation (133). Hanging drop cultures can be obtained by simply dispensing small aliquots of cell suspension (15-25 μl) in a plate, which is subsequently placed upside-down (**Figure 3C**). Gravity enforces cell assembly at the liquid-air interface at the bottom of the hanging drops, and one-spheroid per drop is usually obtained within a few days (133–135). The liquid drops are preserved due to surface tension but evaporation needs to be limited in long-term cultures to maintain appropriate osmolality levels (136).

Tung et al. developed a hanging drop platform for long-term culture and high-throughput analysis (136). Cell suspensions is pipetted into the 384 holes of an array plate, where the hydrophilic surface and gravity favor the formation of the hanging drops. Liquid reservoirs at the edge of the plate and a tray filled with water provides humidity while a plate lid limits evaporation (136). This implementation overcomes some drawbacks of the original hanging-drop method, such as rapid dehydration and laborious sample handling. This platform is commercially available in multiple formats from 3D Biomatrix.

InSphero developed a user-friendly hanging-drop platform compatible with long-term cultures and high-quality imaging. The InSphero culture system follows the 3D Biomatrix design, with a hanging-drop array made of multiple inlets inserted between a lid and a liquid reservoir (137). A distinct feature of the InSphero method is that the formed spheroids are transferred into a ULA-plate (Gravity-Trap ULA plate or Akura plates) by adding liquid on top of the inlets or by centrifugation. The spheroids can be maintained in the ULA plates for long-term culture, where liquid evaporation occurs at slow rate, and media exchange can easily be performed (138). The spheroids are confined in a small area (1 mm-diameter) at the bottom of the well, which is flat and made of thin clear plastic, facilitating live, automated and high-quality imaging (138–140). A similar implementation is also available nowadays from 3D Biomatrix (**Figure 3D**). The 3D Biomatrix and InSphero platforms are compatible with both manual and fully automated handling.

The hanging drop method generates spheroids from a variety of cell types, such as tumor cell lines or cells derived primary tumors or healthy tissue (102, 125, 133). Cell differentiation and extensive secretion of extracellular matrix (133), as well as inner regions of necrosis and hypoxia (141) have been described. Herter et al. obtained heterotypic spheroids of adenocarcinoma cells and fibroblasts (142). The heterotypic spheroid model has been used to evaluate the combinatorial effect of IL-2 variant and tumor- or fibroblast-targeted T cell bispecific antibodies on T, NK and NKT cell activation, cytokine and chemokine secretion, spheroid killing and infiltration (142). Interestingly, the fibroblasts spontaneously arranged in the spheroid core, as also reported by others using different cell types and spheroid culture methods (122, 139, 142). The hanging drop technique has also been applied to immune therapy screening, for instance to test the effect of rituximab on NK cell ADCC in primary follicular lymphoma spheroids (125), and to evaluate different protocols of NK cell activation in colorectal carcinoma models (135). In summary, the hanging drop method is an easy-to-use technique to generate biologically relevant tumor models *in vitro*, it is compatible with multiple end-point assays, live and high-quality imaging, and high-throughput screening. However, the hanging-drop method does not fully address the problem of heterogeneous structural integrity between cell lines, as multiple cases of loose aggregates can be found literature (133).

### 3.4 Scaffold-based spheroid cultures

*In vivo*, NK cells migrate through the supportive tissue, and the stroma composition influences their infiltrative capacity, as well as tumor aggressiveness and therapeutic responses. Similarly, the spheroid stiffness, together with the amount and the composition of the ECM, shapes NK cell activity *in vitro* (112, 115, 143, 144). Therefore, the properties of the ECM should be considered when evaluating NK cell-spheroid interactions.

A strategy to include ECM-like conditions and chemokine gradients in the assays is to introduce scaffolds in the cultures. Examples of such scaffolds are hydrogels or porous inserts that can be obtained from animals (e.g., fibrous gelatin, collagen, Matrigel, chitosan, hyaluronic acid, silk fibroin), plants (e.g., alginate) or by synthetic production (e.g., polyethylene glycol, polylactic acid, poly-ϵ-caprolactone, polyurethans) (24, 25, 28, 145). Scaffolds used in NK cell 3D research are mostly hydrogel-based, and they include Matrigel (95, 144, 146), collagen type I hydrogels (92, 143, 147–149), alginate hydrogel (95, 150) and functionalized hydrogel based on polyethylene glycol (PEG) (151).

Schnalzger el al. established heterotypic cultures of normal and tumor colon organoids together with primary fibroblasts in Matrigel to evaluate the efficacy and the specificity of EpCAM-CAR-NK92 cells (144). Similarly, Gopal et al. tested NK92-CD16 cytotoxicity in combination with chemotherapy and antibody-based immunotherapy against pancreatic and breast cancer cells embedded in Matrigel, using a newly designed high-throughput micropillar-microwell sandwich platform (146).

Ayuso et al. combined the use of hanging drop spheroid formation (scaffold-free method), rat-derived collagen type I hydrogel (scaffold-based method) and microfluidics to study antibody penetration, ADCC, and NK cell chemotactic migration and infiltration in breast cancer spheroids (**Figure 3E**) (92). This microfluidic device consisted of a central chamber filled with collagen type I hydrogel, flanked by two lateral lumens covered with endothelial cells to mimic blood vessels. Hanging drop-derived spheroids were transferred into the central chamber and embedded together with NK cells in collagen, while a solution containing IL-2-conjugated CD16-EpCAM bispecific antibody was perfused through the later lumens. Using this method, the authors could follow the dynamic of antibody penetration, NK cell chemotactic migration and ADCC by imaging (92).

Marrella et al. developed alginate spheres to study the effect of IFN-γ exposure on NK cell ligand expression in neuroblastoma spheroids (150). The same group combined the use of alginate spheres with the MIVO fluidic platform (93, 94) to study the mechanisms of NK cell extravasation and tumor infiltration (**Figure 3F**) (95). The MIVO platform consists of a trans-well system connected to a closed-loop fluidic circuit (93, 94). The top chamber of the trans-well (donor chamber) contained alginate-derived neuroblastoma spheroids. NK cells were constantly perfused through the bottom chamber (receiver chambers) and the capillaries of the fluidic circuit, which mimics the blood circulation. The porous membrane physically separating the donor and the receiver chambers prevented alginate spheres to fall into the receiver chamber (95). The spheroids and the cell suspension could be recovered and used for a variety of end-point analysis. For instance, the authors used flow cytometry to compare the proportion of CD16^+^ CD45^+^ cells in circulating, extravasated and spheroid-infiltrated NK cells (95).

Animal- and plant-derived hydrogels, such as collagen and Matrigel, present some limitations, such as batch-to-batch variability, long-term instability, cell degradation and immunogenicity, therefore synthetic hydrogels might be preferred for some applications (28). For instance, Temple et al. developed arginine-glycine-aspartic acid functionalized PEG hydrogels to study the effect of matrix metalloproteinases and integrins on NK cells migration (151). The authors also showed decreased NK cell infiltration in non-small lung cancer 3D cultures releasing soluble MICA and TGF-β (151).

The scaffold-based spheroid cultures are biologically relevant systems to study leukocyte transmigration and tissue infiltration, however they are often not compatible with high-throughput screening and live cell imaging. This, combined with the fact that the procedures to recover cells from the hydrogels are quite laborious, limits their large-scale application, and it is one of the reasons why scaffold-free technologies are still usually preferred.

### 3.5 Miniaturized platforms for spheroid studies

Another strategy to enhance the interplay of spheroid and NK cells is the use miniaturized cell culture platforms. In miniaturized platforms, the well dimensions are tailored to the spheroid dimension, ensuring physical confinement, facilitating cell detection and cell-to-cell interaction. The miniaturization scales down the volumes and cell number required to set up and maintain cultures, enabling spheroid formation when the material is scarce (as with many patient samples). We already described a few examples of scaffold-based microfluidic and miniaturized platforms in the previous paragraph (146, 148). Here, we will focus on scaffold-free miniaturized spheroid cultures for NK cell research.

Nguyen et al. designed a miniaturized microfluidic platform to enhance NK interaction with tumor and cardiac spheroids and study the specificity of NK cell killing and the secondary effects on healthy tissue (152). Their microchip, adapted from the Akura Flow MPS discovery platform (153), features two individual perfusion channels with 10 communicating compartments and medium reservoirs at both ends (152, 153). Spheroids were formed in ULA plates and then transferred into the compartments, and NK cells were introduced to the cultures through the medium reservoirs. Perfusion across the medium reservoirs and the spheroid compartments, and therefore NK-spheroid interaction, was driven by gravity simply tilting the microchip along its long axis. The process is automated using a tilting stage (152, 153). The authors showed the relevance of the method by perfusing umbilical cord blood (UCB)-derived NK cells into colorectal tumor spheroids and cardiac microtissues placed in distinct communing compartments. The experiment demonstrated the specificity of UCB-NK cell cytotoxicity towards colorectal tumor spheroids, but it also showed the presence of infiltrating UCB-NK cells in the cardiac spheroids along with signs of arrhythmia, possibly induced by pro-inflammatory cytokines detected in the supernatant (152). Cells and culture medium can easily be recovered by pipetting for cellular and proteomic analysis, while end-point imaging can be performed directly on the chip. However, dynamic of NK cell adhesion and infiltration cannot be entirely followed by live imaging, due to the tilting procedure, and the spheroid culture cannot be performed directly on the microchip.

Our group has developed a scaffold-free microwell chip platform to study the dynamics of NK cells in tumor spheroids (154–156). One of the main advantages of this platform is that all the experimental phases (spheroid formation, treatment and NK cell incubation, analysis) can be performed on the chip, which is compatible with high-resolution and live cell imaging. Spheroids and NK cells can also be retrieved by standard pipetting for off-chip analysis (155). The spheroid formation is obtained by seeding cell suspensions in a silicon microwell array chip covered with a non-adhesive coating and inducing cell aggregation by ultrasonic standing waves (USW) (**Figure 3G**) (155, 156). In microfluidics and other miniaturized systems, ultrasound has found use in various applications where either suspended cells or the fluid is manipulated by acoustic radiation pressure. This technology field is often called microscale acoustofluidics (157). We specifically use this technology to induce cell aggregation and formation of single, homogeneous and compact spheroids in each well (154, 158–161). When the USW is turned off, cellular production of ECM proteins and formation of tight intercellular connections continue, enabling long-term spheroid cultures. Importantly, acoustic trapping is also performed while setting up the co-cultures, to induce NK cell-spheroid interaction (156, 161). Importantly, the physical force field of the USW is gentle to cells even at long continuous exposures (several days) (157).

Different microwell chip designs have been developed during the years (155, 156, 159, 161). The most recent is a multichambered microwell chip, with 16 compartments each containing 36 microwells, allowing the simultaneous production and analysis of 576 spheroids (36 spheroid replicates for each experimental condition) (156). The microwell array is made in silicon, which is bonded to a glass bottom layer that is compatible with high-quality imaging. A thin and transparent non-adhesive coating is applied at the bottom of the wells to prevent cell adhesion (162). A PDMS frame separates the wells into compartments serving as liquid reservoirs (typically 50 μl are used for each compartment). Contamination and evaporation are prevented by placing a coverslip on top of the PDMS frame (156).

Fast image acquisition combined with high-resolution imaging enables high-throughput screening at chamber levels with detailed analysis of biological processes at the single microwell level. The method allowed studies of how combinatorial treatments affect NK cell infiltration and activity in tumor spheroids, quantifying the amount and the timing of killing (155). The method is also compatible with the formation of a wide range of homotypic spheroids (kidney, thyroid, ovarian, hepatic, non-small lung cancer cell carcinomas) (155, 156, 161), heterotypic and patient-derived spheroids (unpublished results), the latter particularly facilitated by the low material requirement.

The main limitations of the miniaturized platforms described here are related to the number of conditions that can be tested in parallel and the number of cells that can be retrieved from the chips for further analysis.

In this section, we have described the methods used for spheroid formation in the field of NK cell research. Each technique presents advantages and limitations, which should be carefully evaluated when choosing experimental strategy. The choice of method to use should be driven by two main questions: Which method provides the most relevant 3D features to study the mechanisms of interest? Which method is compatible with the analysis/assays we want to perform? In the next section, we will describe the available assays to characterize NK cell behavior and phenotype in cancer spheroids.

## 4 Methods to study NK cell activity in tumor spheroids

The discovery of novel cell types and their characterization is closely related to our ability to detect their function. As a matter of fact, various immune cell types have been identified through observation of their activity, which historically preceded their phenotypic characterization. This is also the case for NK cells, initially identified for their ability to “spontaneously kill” tumor cells *in vitro* (40, 163, 164), and later characterized for their serial killing capacity in single cell screening assays (165–167). A variety of well-established assays and cutting-edge technologies are routinely used to screen NK cell activity on tumor cell monolayers (165, 167–174). However, a limited number of them is suitable for studying NK cell cytotoxicity in tumor spheroids.

Compared to 2D systems, developing functional assays for 3D cultures is more demanding. The challenges are intrinsically related to the physical properties of the spheroids. Accurate imaging of 3D objects beyond ≈50 μm is challenging. High cellular density and ECM deposition generate light scattering, limiting the detection of NK cytotoxicity in the inner areas of the spheroids. Therefore, enzymatic dissociation or tissue sectioning are performed to isolate and characterize infiltrating NK cells, but these processes cause loss of spatial information and dynamics. In addition, introducing sample processing steps slows down the analysis and might cause experimental artifacts. Consequently, very few technologies that combine high-throughput cytotoxicity screening and high-resolution analysis of killing mechanisms are available, and multiple methodologies might be required to fully dissect NK cytotoxicity in tumor spheroids.

A lot of research is currently undergoing to overcome these limitations. In this section, we describe the methodologies described in literature.

### 4.1 Bulk assays or methods based on radioisotope-cell labeling and luminescence

Traditionally, Chromium-51 (^51^Cr) release assay has been the gold-standard method to quantify NK cell cytotoxicity against tumor cell suspension or monolayers (175). The procedure involves culturing NK cells with tumor cells pre-labeled with sodium chromate. NK cell cytolytic activity causes loss of tumor cell membrane integrity, and consequent release of ^51^Cr radioisotope in the supernatant, which can be detected by either γ or β counters. Thus, quantification of the released ^51^Cr is a measurement of NK cell cytotoxicity. Being quantitative, ^51^Cr release assay is particularly adapted to compare the susceptibility of different cell types to various immunotherapeutic treatments (175).

^51^Cr release assay has also been used to study NK cell cytotoxicity against tumor spheroids (115, 129, 130, 176), coupling the conventional quantification of killing with the identification of dead areas by autoradiography (115). While ensuring high sensitivity, the use of radioactive material also represents the main limitation of the method. Multiple precautions should be taken while using ^51^Cr to ensure the staff safety and proper waste disposal, and license to work with radioactivity must be granted. This limitation motivated the development of colorimetric and luminescence assays to measure the release of non-radioactive intracellular contents, such as lactate dehydrogenase (LDH) and adenosine 5´-triphosphate.

LHD is a cytosolic enzyme that catalyses the conversion of lactate to pyruvate via NAD^+^ reduction to NADH. NADH is a used by the reductase as co-factor to catalyze reduction reactions. A typical strategy to measure the LHD released in the supernatants of NK and spheroid co-cultures is to use a mix containing the reductase, its substrate, together with lactate and NAD. For instance, the LHD assay has been used by Murphy et al. to show the importance of addressing hypoxia to improve the efficacy of cell therapy (110). To increase the oxygen supply in the spheroid core, they developed biocompatible poly(lactic-co-glycolic) manganese dioxide nanoparticles (PLGA-MnO_2_ NPs) that catalyse oxygen production from tumor-derived hydrogen peroxide. By treating hypoxic breast cancer spheroids with PLGA-MnO_2_ NPs, they demonstrated a decreased presence of HIF-1α^+^ tumor cells in the core associated with a significant reduction of immunosuppressive factors such as adenosine and lactate production. This phenotype was accompanied by an increased NK cell-mediated cytotoxicity and IFN-γ production in the treated spheroids (110).

Bulk methods are high-throughput, therefore good for screening NK activity against multiple targets and combinatorial treatments. However, they provide no information about cell phenotype, neither the kinetics and the localization of NK cell killing and infiltration. Therefore, they are mainly used as end-point assays for screening multiple conditions, sometimes used as a guide for finding the best experimental conditions and performing more detailed analysis by flow cytometry or imaging.

### 4.2 Flow cytometry of tumor spheroids

Flow cytometry provides a rapid, quantitative and multi-parametric analysis of single cells. Applications of standard flow cytometry include identification of cell types based on lineage markers, quantitative expression of membrane-bound and intracellular molecules, and analysis of cell status and function. The multi-parametric power of flow cytometry mostly derives from the ability to collect and differentiate multiple fluorophores. Depending on the number of lasers, detectors and filter combinations, the most recent configurations of commercially available flow cytometers can discriminate more than 30 different emitting fluorophores (177–179). Flow cytometers can detect cells and particles within 0.2-150 μm in diameter, although the use specialized systems can allow the detection outside this range.

Flow cytometry is frequently applied to spheroid research as end-point assay to quantify NK cell infiltration, characterize the viability, proliferation and phenotype of NK cells and tumor cells isolated from spheroid cultures, and to analyze functional parameters, such as degranulation and cytokine production of spheroid-infiltrating NK cells. A strategy to separate spheroid-infiltrating NK cells from extra-tumoral NK cells is to collect the culture supernatant (extra-tumoral NK cell fraction) and the spheroid mass (spheroid-infiltrating NK cell fraction) in different tubes prior to tissue processing and immunostaining (106, 112, 119, 180). Flow cytometry analysis of the two fractions showed enrichment of NK cells and CD8^+^ T cells in the spheroid core (112, 119), demonstrating a better infiltrative ability of these two populations compared to CD4^+^ T cells, further enhanced by IL-15 activation (119).

Similar to what has been observed in tumor patients (118), spheroid-infiltrating NK cells tend to lose the expression of NKG2D and DNAM-1 (119, 124). NK2GD expression could be partially restored by blocking MICA/B, contributing to better NK cell cytotoxicity and infiltration (119). The levels of soluble NKG2D ligands are high in the plasma obtained from tumor patients, and their levels tend to decrease after surgery, further proving their implication in cancer progression. In line with that, Giannattasio et al. showed abundant shedding of NKG2D ligands in cervical carcinoma spheroids, associated with decreased expression of the membrane-bound form (106). Increased HLA class I expression has also been observed in tumor spheroids compared to monolayer cultures (181).

NK cell cytotoxicity can be analyzed by flow cytometry calculating the percentage of dead tumor cells, identified with apoptotic markers and/or cell impermeant dyes. Combinatorial staining with Annexin V and 7-AAD or propidium iodine staining is frequently used to quantify early and late apoptosis of spheroid-derived tumor cells and NK cells (119, 125, 180, 182, 183). Veneziani et al. performed flow cytometry analysis of patient-derived neuroblastoma spheroids co-cultured with NK cells and Nutlin-3, characterizing the phenotype of NK cells together with the apoptotic state of tumor cells. They demonstrated up-regulation of ULBPs, PVR and Nectin-2 on Nutlin-3-treated tumor cells, associated with increased NK cell cytotoxicity (183). NK cell functionality can also be inferred by qualitative and quantitative assessment of cytokine production and granule content (IFN-γ, TNF-α, MIP-1α, perforin, granzyme B) and granule release (CD107a).

The main advantage of flow cytometry is the multi-parametric analysis of single cells at high-throughput. Despite the considerable amount of information that can be retrieved, flow cytometry lacks spatial and temporal resolution. Additionally, it can introduce technical artifacts. Such risk can be reduced by using mild dissociation agents and decreasing the time between the dissociation and the flow cytometry acquisition. NK cell killing over time can only assessed by preparing multiple NK-tumor co-cultures and analyzing them at different timepoints, with technical variability affecting the robustness of the assay. Therefore, it would be preferable to use flow cytometry for a single-timepoint experiment and move to other techniques when spatial information and dynamics are part of the biological question.

### 4.3 Measuring NK cell cytotoxicity by detecting spheroid physical changes

NK cell activity can cause variations of the spheroid physical properties, such as diameter, volume, and weight, which can be detected and quantified to estimate NK cell-mediated spheroid killing (117, 119, 127, 142, 183). Rademacher et al. monitored the diameter of sarcoma spheroids over time by widefield microscopy to study the effect of IL-12 on NK92 cytotoxicity and infiltration. They detected a reduction of spheroid diameter incubating NK92 cells with IL-12-engireed osteosarcoma cells, suggesting a positive effect of IL-12 on NK cell activity in 3D (127). Similarly, the reduction of spheroid volume was used to evaluate the benefits of cytokine activation on PBMC cytotoxicity against heterotypic and homotypic colorectal cancer spheroids (119, 142). Sargenti et al. developed a fluidic platform to estimate NK cell cytotoxicity and infiltration measuring spheroid weight and diameter (117). The method is based on tracking the motion of spheroids falling into a vertical flow channel using a brightfield imaging system (117, 184). Analyzing spheroids obtained from four different colorectal tumor cell lines, the authors were able to correlate the mass density with the degree of spheroid compactness (117). Co-incubation with NK cells led to a significant reduction of spheroid weight and diameter, while a temporary increase of spheroid mass density was associated to NK cell infiltration (117). There are reports saying that spheroid volume does not affect NK cytotoxicity (97, 115), while spheroid cellular density and compactness do (115, 117). The analysis of spheroid physical properties represents a fast, non-invasive and easily accessible method to measure NK cell cytotoxicity. However, its application is arguably limited, due to lack of sensitivity and information. During early phases of NK killing, spheroids often get partly disintegrated and less compact, leading to increased volume which may seem contradictory. In addition, NK cell infiltration itself might induce volume and mass changes, which are difficult to isolate and subtract from the quantification. The method itself does not provide information regarding the mechanisms of NK cell killing and spheroid death. To overcome these limitations, the study of spheroid physical properties is usually combined with histological characterization and other cell labeling-based quantitative analysis.

### 4.4 Measuring NK cell cytotoxicity and infiltration by detecting spheroid histological changes

A method to characterize NK cell cytotoxicity and infiltration in the spheroids is by performing histochemical staining of sections. Various histological changes have been associated with NK cell activity in tumor spheroids, such as loss of surface integrity (98, 115), formation of cytoplasmic blebs, chromatin condensation (111, 116) and mitochondrial swelling (98). Simply using immunostaining and haematoxylin/eosin counterstaining, Kaaijk et al. described two modalities of LAK killing in glioma spheroids: a) apoptosis, characterized by loss of cell volume, chromatin condensation and formation and apoptotic bodies; b) necrosis, identified by loss of fibrillary structure and acquisition of smooth appearance in the cytoplasm, loss of membrane integrity and swollen nuclei (116). Studying the relative position of LAK and dead glioma cells in spheroid tissue sections, Jääskeläinen et al. localized tumor cell death in areas devoid of LAK infiltration, speculating that contact-independent killing modalities mediated by soluble factors could play a role (111). Contact-mediated killing has been demonstrated by Iwasaki et al. imaging ultrathin spheroid sections by transmission electron microscopy (98). Using this technique, the authors captured the formation of tight cytoplasmic interdigitations between effector and target cells in the spheroid core, and the development of intracytoplasmatic dense granules that usually precedes degranulation (98).

To introduce the temporal factor, it is possible to collect and stain spheroids at different times, to follow the progressive infiltration and cytotoxicity of NK cells toward the core (98, 112, 115). To quantify this behavior, there have been multiple attempts to classify NK cell infiltration or/and spheroid death (98, 105). For instance, Jääskeläinen et al. calculated the density of LAK cells in three different spheroid areas, corresponding to the periphery (100 μm depth from the spheroid surface), the intermediate layer (100 μm to 200 μm depth) and the core (200 μm to 300 μm depth) to quantify the involvement of adhesion molecules on LAK infiltration. According to their findings, the expression of adhesion molecules varied in the different areas and it was strongly influenced by the secretory activity of LAK cells. For instance, the levels of CD54 were weak in periphery of glioma spheroids and intense in the intermediate rim in the absence of LAK cells, while the expression intensified along the frontier of migrating cells possibly due to IFN-γ release. Blocking CD54 completely prevented LAK migration into the spheroids, showing the relevance of this pathway for NK infiltration (112).

Iwasaki et al. classified the activity of LAK cells into four categories based on both infiltration and cell damage: grade I) effectors in contact with the spheroid surface and little target death; grade II) effector infiltration into the outer third layer of the spheroid accompanied by target cell death; grade III) target death detected in the middle layer of the spheroid; grade IV) target death detected in the core of the spheroid (98). Garcia de Palazzo et al. developed a similar system to classify the histological changes of colon cancer spheroids and quantify the effect of CA19-9-CD16 bispecific antibody on LAK 3D killing (105). Based on haematoxylin/eosin staining, they classified the histological damage into five grades calculating the percentage of necrosis in relation to the control condition (105).

Histochemistry can be also applied for the analysis of NK cell status in the TME. For instance, Weil et al. detected apoptotic NK cells in the core of head and neck squamous cell carcinoma spheroids following soluble MICA exposure (118), demonstrating the detrimental effect of soluble NKG2D ligands on NK cell-mediated tumor surveillance (118, 185).

Despite these efforts, the use of spheroid morphological changes to quantify NK cytotoxicity remains problematic, suffering from subjective evaluation and lack of universal classification. Lack of three-dimensionality and time resolution represent additional limitations. Tissue reconstruction could retrieve 3D spatial information, but it is technically difficult and rarely performed. The kinetics of NK cytotoxicity can be studied performing time-course assays on spheroid replicates, however limited time resolution (usually day-scale) and sample availability makes live cell imaging a preferred option, which is also compatible with cell tracking. Despite these limitations, histological analysis is considered a valuable and very informative option as end-point assays and qualitative analysis, especially to confirm data obtained by other techniques (119, 135). The recent development in the field, such as the release of methodology and machines for automated multiplexed staining and analysis (e.g. Hyperion, MACSima, CODEX), will most likely increase the use of this technique to NK cell and spheroid studies.

### 4.5 Measuring NK cell cytotoxicity and infiltration using fluorescence microscopy

A variety of fluorescent dyes have been developed to characterize cell status, such as viability, apoptosis, necrosis, proliferation, and metabolism. If properly chosen, these markers can provide information on the killing modality. For this reason, coupling cell labeling with fluorescence-based imaging techniques is a particularly suitable strategy to characterize NK cell cytotoxicity in tumor spheroids.

The workflow involves cell labeling before, during, or at the end of the cytotoxicity assay, and detection by the appropriate microscopes, such as widefield, confocal, light sheet or two-photon fluorescence microscopes. The variation of the fluorescence intensity during NK-spheroid co-cultures can be quantified, such as loss of viability and proliferation markers, or acquisition of necrotic/apoptotic markers, providing an unbiased evaluation of NK cell activity. In addition, live cell imaging can be performed to study the kinetic of spheroid death and NK cell infiltration with good temporal resolution.

Giannattasio et al. followed the infiltration of Hoechst-labeled NK cells into CFSE-labeled cervical carcinoma spheroids for 48 hours with 30 minutes time resolution using widefield fluorescence microscopy, showing NK cell proliferation and accumulation at the periphery of the spheroids (106). To characterize the spheroid inner areas, imaging techniques with 3D resolution, such as confocal, light sheet and two-photon microscopy, can be easier than sectioning. These techniques provide spatial information of NK cell infiltration and killing while preserving sample integrity. An additional benefit is low sample consumption, as time courses and 3D characterization can be performed simultaneously on a single sample. Imaging a single focal plane inside the breast cancer spheroids with 30 second resolution, Ayuso et al. tracked NK92 chemotaxis towards the spheroid core (92). Using this strategy, they were able to describe the directionality and the modality of NK cell migration, capturing NK cell bodies squeezing between tumor cell junctions to reach the inner areas of the spheroid (92). They quantified NK cell infiltration and spheroid killing, showing that increasing effector-to-target ratios positively influenced NK cell ability to kill multiple spheroid layers (92, 106, 123, 135).

Hoogstad-van Evert et al. analyzed the activity of hematopoietic stem and progenitor cells (HSPC)-NK cells on ovarian cancer spheroids using a similar approach (123). They performed live cell confocal imaging on a single focal plane to study the dynamics of HSPC-NK cell cytotoxicity over five hours. As an end-point assay, they imaged multiple focal planes to collect data from a 60 μm spheroid section, which allowed them to quantify dead tumor cells in relation to tissue depth (123).

*In vitro*, NK cell killing of tumor cell suspensions manifests within a few minutes from stimulation, and it persists for few days before NK exhaustion and/or dysfunctionality occurs. The killing mechanisms can shift over time, as well as tumor cells susceptibility. For these reasons, long-term assays are usually considered particularly appropriate for the characterization of NK cell killing modalities. The duration of the assay is particularly important in 3D, where the infiltration of NK cells and the diffusion of soluble factors influence the killing dynamics. Phototoxicity and photostability are two important parameters to take into consideration while performing long-term imaging assays, as both could introduce technical artifacts and reduce the test sensitivity. For these reasons, internal controls should always be run to test the phototoxicity levels, and dyes resistant to photobleaching and cell-mediated degradation are recommended. If phototoxicity and photostability are properly addressed, long-term imaging assays could be very informative.

**Figure 4:**
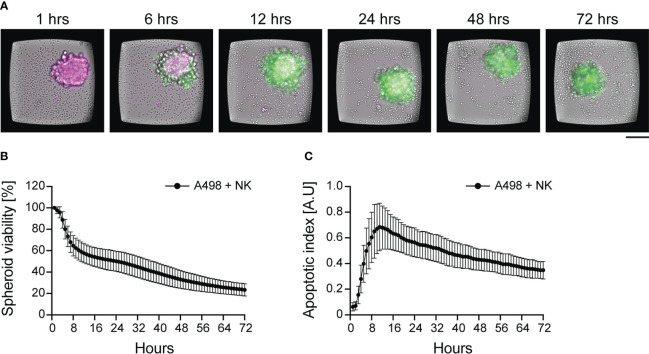
Dynamics of NK cell cytotoxicity against tumor spheroids. IL-15 activated NK cells were incubated with pre-formed renal carcinoma spheroids and imaged for 72 hours. **(A)** Time-lapse sequence of NK cell killing of renal carcinoma spheroids analyzed by live imaging. TMRM (in magenta) and a caspase-3/7 activity reporter (in green) were used to detect spheroid viability and apoptosis, respectively. NK cells can be seen in the brightfield channel. Scale bar: 100 μm. **(B, C)** Time-course of spheroid viability **(B)** and apoptotic index **(C)** from a single microwell chip chamber (n=36). The data were presented in Carannante et al. (186).

Our own time-course analysis revealed high inter-donor heterogeneity in terms of killing dynamics, and it predicted donor-specific long-term and short-term response to combinatorial treatment *in vitro* (**Figure 4**) (156, 186). We tracked NK cell-mediated cytotoxicity of renal and ovarian cancer spheroids for three days combining the use of two fluorescent dyes: a mitochondrial activity reporter (TMRM) as viability marker, and a caspase-3/7 activity reporter as apoptotic marker (**Figure 4A**). As expected, NK cell activity induced a cumulative loss of spheroid viability over time (**Figures 4A, B**). However, a detailed characterization of the time-courses showed high variation in killing dynamics and long-term response to combinatorial therapy among NK donors. In the same assay, the analysis of caspase-3/7 intensity curves revealed the time of maximum NK cell-mediated apoptotic death, corresponding to the peak of fluorescent intensity. In addition, the combined used of two dyes allowed us to normalize the amount of apoptotic death for the initial spheroid viability, providing a more sensitive parameter for comparing different spheroid types (**Figure 4C**). Thanks to the long-term stability of the dyes, we were able to perform 3D confocal microscopy at the end of the live imaging assays to localize tumor cell apoptosis (156). Knowing when NK cell populations reach their maximum activity could be valuable information for designing personalized combinatorial therapy. It could also reveal the presence of different NK populations active at different times.

Courau et al. combined long-term live imaging with flow cytometric end-point analysis to study the contribution of each PBMC population to spheroid cytotoxicity and infiltration (119). They observed infiltration and killing of colorectal cancer spheroids under IL-15 exposure, mainly driven by NK and CD8^+^ T cells. The presence of activated PBMCs induced HLA-expression on tumor spheroids, while infiltrated NK cells showed a reduction of NKG2D expression. Coupling anti-MICA/B antibody with anti-NKG2A checkpoint blockade enhanced spheroid apoptosis and PBMC infiltration (119).

If multiple set of assays are planned, it is a common practice to genetically modify tumor cells to stably express fluorescent proteins (120, 123, 125, 135, 144, 180, 187). This strategy allows direct assessment of spheroid viability by measuring the variation of fluorescence intensity, removing the need of additional staining steps. For instance, Susek et al. applied this strategy to study the efficacy of chimeric switch receptors (CSR) for cell-based immunotherapy (120). To revert PD-1-mediated NK cell inhibition, they designed a PD-1-CSR replacing the inhibitory intracellular domains of PD-1 with activating motifs. They quantified the activity of PD-1-CSR NK-92 cells against renal carcinoma spheroids expressing red fluorescent protein by live imaging, demonstrating good specificity and cytotoxic activity of the cell product (120). Lanuza et al. used EGFP-transfected colorectal carcinoma cells to facilitate the detection of the spheroid area, used to quantify the cytotoxic effect of different effector-to-target ratios over time (135).

**Figure 5:**
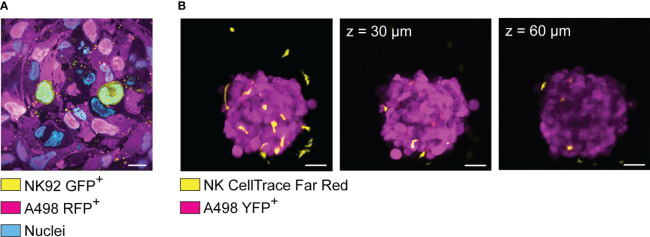
Visualization of spheroid-infiltrating NK cells by light sheet microscopy and confocal microscopy. **(A)** Light sheet microscopy image showing NK92 cells (yellow) infiltrating a renal carcinoma spheroid (magenta). RFP^+^ A498 renal carcinoma spheroids were incubated with GFP^+^ NK92 cells for 2 hours before undergoing tissue expansion in deionized water and imaged by light sheet microscopy. **(B)** YFP^+^ A498 renal carcinoma spheroids were incubated with resting NK for 48 hours before being imaged by confocal microscopy. NK cells were detected in both extra-tumoral and intra-tumoral areas. However, the signal was progressively lost at increased depth. Left panel: 3D rendered confocal stack of an A498 renal carcinoma spheroid (magenta) co-cultured with NK cells (yellow). Central panel: optical section of the spheroid showed in the left panel (z = 30 μm). Central panel: optical section of the spheroid showed in the left panel (z = 60 μm).

We used GFP-transfected NK92 to visualize the formation effector-to-target cell contacts in renal carcinoma spheroids using light sheet microscopy (**Figure 5A**) (188). Särchen et al. combined the use of fluorescently labeled proteins with cell death markers to calculate spheroid killing as the ratio between the two parameters, allowing a fair comparison between spheroids heterogeneous in size. Using this method, they showed the positive effects of BH3 mimetics on NK cell killing of pediatric cancer spheroids (187). Cell transfection with fluorescent proteins presents multiple advantages, such as reducing the optimization steps and facilitating the analysis. However, this approach is not suitable for all types of cells and applications since the transfection efficiency varies among cell types, with some being very resistant to genetic modifications, and cell manipulation is not compatible with the characterization of primary tumor samples, such as patient-derived tumor spheroids, since it might cause loss of the original sample features.

Apart from choosing the right labeling strategy, phototoxicity and photostability could be minimized by choosing microscopy techniques particularly suited for live 3D imaging, such as light sheet microscopy instead of confocal microscopy (189). Confocal microscopes involve a single objective for both illumination and detection, and 3D resolution is achieved generating signal from out-of-focus planes, which is then filtered out to detect the focal plane of interest. This design leads to relatively high photobleaching and phototoxicity, while sacrificing fluorescent signal to achieve optical sectioning. However, smaller spheroids can be imaged live with good results by confocal microscopy for a limited time, for instance to quantify NK cell infiltration (see example in **Figure 5B**). In light sheet microscopes, two separate objectives are used for illuminating the sample and detecting the signal. The illumination objective focuses a sheet of light to the plane of interest, and the detection objective collects light from the excited focal plane. Using this design, only the focal plane of interest is illuminated, causing little photobleaching and phototoxicity. Since the whole focal plane is imaged, the acquisition is dramatically faster than point-scanning confocal imaging (190, 191). These features make light sheet microscopy particularly suitable for imaging fast biological events in thick samples, such as cell division and NK cell infiltration in tumor spheroids (192). Thick samples scattering large amount of light can be imaged using multi-view acquisition, i.e., imaging the sample from multiple angles and combining the information during post-processing (190). This feature has been used by Del Bano et al. to study the effect of anti-mesothelin/CD16 bispecific antibody on NK cell infiltration in triple negative breast cancer spheroids (109). We combined cell transfection with fluorescent proteins, light sheet microscopy and tissue expansion to achieve detailed visualization of spheroid-infiltrating NK cells (**Figure 5A**) (188). Specifically, RFP^+^ renal carcinoma spheroids were incubated with GFP^+^ NK92 cells for 2 hours before undergoing tissue expansion and light sheet microscopy imaging. This technique is suitable for imaging the details of NK cell interactions with tumor cells in thick samples (188), for instance to obtain snapshots of killing mechanisms, metabolic activity, or receptor modulation. Like every technique, also light sheet microscopy presents its own disadvantages. The sample is commonly embedded in hydrogel, and some embedding media are not compatible with all cell types. The embedding procedure can be quite laborious, it can affect sample viability, and it is not compatible with high-throughput analysis. Finally, a typical light sheet microscopy experiment generates large amount of data, which are time consuming to transfer and process, and require high computational and storage capacity.

## 5 Conclusions

Since immunotherapy became a standard clinical practice, multiple cases of innate and acquired resistance have been reported, pointing out that more research is needed to understand the mechanisms of immunosuppression and to predict individual responses to treatment. To gain more knowledge, we need robust *in vitro* systems suitable for immuno-oncology studies. The characterization of NK cells in the original tumor tissues is challenging due to limited infiltration and tumor-driven changes in their phenotype (193). 3D cultures represent a fantastic tool to overcome these issues. The application spans from identifying the reasons behind poor NK cell performance in solid tumors, to developing and testing new strategies to boost their activity. The 3D platforms currently available allow analysis of cell phenotype, spatial distribution, and function. Phenotype, localization, and function can be analyzed at different levels, from gene expression to cell morphology and dynamics. Despite the broad range of characterization that they allow, their full potential is rarely exploited. In NK cell research, tumor spheroids are still mainly used as “support assays” to confirm data obtained from 2D assays or *in vivo*. The main application remains testing NK cells in combination with other therapies, most of the time without providing a full characterization of neither NK cells nor tumor spheroids. It is not rare to find qualitative data of NK cell infiltration and killing with no quantification provided. The main challenges are to obtain high-throughput, high-quality and quantitative imaging, as well as biologically relevant models of the solid tumor microenvironment. In this regard, rigorous validation of the model should be performed by comparing the 3D architecture and the cellular composition of the original tissue with the *in vitro* 3D culture, following its evolution over time. This is particularly important for NK cell research, considering the impact that tissue architecture, cellular and extracellular composition has on immune cell migration and cytotoxic capability. More developed use of 3D cultures combined with automated analysis pipelines, perhaps artificial intelligence-driven, could expand their application to NK cell mechanistic studies and quantitative analysis. Still, more research is needed to fully understand and exploit the possibilities that 3D cultures can offer.

## Author contributions

VC and BÖ drafted the content of the manuscript. VC wrote the first draft of the manuscript that was edited by to its final form by VC, MW, and BÖ. All authors contributed to the article and approved the submitted version.
